# Germline Genetic Risk Variants for Progressive Multifocal Leukoencephalopathy

**DOI:** 10.3389/fneur.2020.00186

**Published:** 2020-03-17

**Authors:** Peggy S. Eis, Christopher D. Bruno, Todd A. Richmond, Igor J. Koralnik, Barbara A. Hanson, Eugene O. Major, Christina R. Chow, Houria Hendel-Chavez, Bruno Stankoff, Jacques Gasnault, Yassine Taoufik, Eli Hatchwell

**Affiliations:** ^1^Population Bio, Inc., New York, NY, United States; ^2^Emerald Lake Safety LLC, Newport Beach, CA, United States; ^3^Richmond Bioinformatics Consulting, Seattle, WA, United States; ^4^Department of Neurology, Feinberg School of Medicine, Northwestern University, Chicago, IL, United States; ^5^Laboratory of Molecular Medicine and Neuroscience, National Institute of Neurological Disorders and Stroke, National Institutes of Health, Bethesda, MD, United States; ^6^Department of Hematology and Immunology, Hôpitaux Universitaires Paris-Sud, INSERM 1184, Faculté de Médecine Paris-Sud, Le Kremlin-Bicêtre, France; ^7^Department of Neurology, Hôpital Saint-Antoine, Paris, France; ^8^Department of Internal Medicine, Hôpitaux Universitaires Paris-Sud, Le Kremlin-Bicêtre, France; ^9^Population Bio UK, Inc., Oxfordshire, United Kingdom

**Keywords:** genetic risk, immunodeficiency, JC virus, multiple sclerosis, natalizumab, progressive multifocal leukoencephalopathy, PML, serious adverse event

## Abstract

Progressive multifocal leukoencephalopathy (PML) is a rare demyelinating disorder of the brain caused by reactivation of the JC virus (JCV), a polyomavirus that infects at least 60% of the population but is asymptomatic or results in benign symptoms in most people. PML occurs as a secondary disease in a variety of disorders or as a serious adverse event from immunosuppressant agents, but is mainly found in three groups: HIV-infected patients, patients with hematological malignancies, or multiple sclerosis (MS) patients on the immunosuppressant therapy natalizumab. It is severely debilitating and is deadly in ~50% HIV cases, ~90% of hematological malignancy cases, and ~24% of MS-natalizumab cases. A PML risk prediction test would have clinical utility in all at risk patient groups but would be particularly beneficial in patients considering therapy with immunosuppressant agents known to cause PML, such as natalizumab, rituximab, and others. While a JC antibody test is currently used in the clinical decision process for natalizumab, it is suboptimal because of its low specificity and requirement to periodically retest patients for seroconversion or to assess if a patient's JCV index has increased. Whereas a high specificity genetic risk prediction test comprising host genetic risk variants (i.e., germline variants occurring at higher frequency in PML patients compared to the general population) could be administered one time to provide clinicians with additional risk prediction information that is independent of JCV serostatus. Prior PML case reports support the hypothesis that PML risk is greater in patients with a genetically caused immunodeficiency disorder. To identify germline PML risk variants, we performed exome sequencing on 185 PML cases (70 in a discovery cohort and 115 in a replication cohort) and used the gnomAD variant database for interpretation. Our study yielded 19 rare variants (maximum allele frequency of 0.02 in gnomAD ethnically matched populations) that impact 17 immune function genes (10 are known to cause inborn errors of immunity). Modeling of these variants in a PML genetic risk test for MS patients considering natalizumab treatment indicates that at least a quarter of PML cases may be preventable.

## Introduction

Progressive multifocal leukoencephalopathy (PML) is a rare CNS disorder that typically occurs in the context of immunosuppression. Examples include HIV infection, hematological malignancies, or following immunosuppressive treatments for autoimmune diseases such as multiple sclerosis (MS) or transplantation (1–3). Infection with JC virus (JCV), a polyomavirus that is common in the general population, is a prerequisite (4). Diagnosis can be challenging as at least 20% of patients can repeatedly and falsely test negative for JCV DNA in cerebrospinal fluid (CSF) (5, 6), one of the main criteria for diagnosis (7). Furthermore, patients are frequently asymptomatic for months and studies indicate that more frequent MRI monitoring could help with early diagnosis and improve prognosis (5, 8), which would be particularly important to implement for high risk patients.

Increased risk of PML in patients on immunosuppressant therapies is well-documented, with the MS/Crohn's disease drug natalizumab carrying the highest risk (3, 9). Other MS drugs with PML risk are fingolimod and dimethyl fumarate (9, 10). Rituximab, which is used to treat a variety of conditions such as hematological malignancies, systemic lupus erythematosus (SLE), and rheumatoid arthritis (RA), has been implicated as a cause of PML for several years but it has been difficult to establish its level of risk (11–14).

The only therapeutic approaches that showed some efficacy in the control of JCV replication in the CNS are based on immune restoration. Depending on the underlying disease before onset of PML, ~20–90% of patients die and survivors develop severe physical and cognitive disabilities (15, 16). Due to the severity of PML as a serious adverse event (SAE) linked to several immunosuppressant drugs, an ideal scenario would be to administer a PML risk test and exclude high risk patients from a therapy in order to prevent PML cases. Currently, there are no highly effective PML risk prediction tests in clinical practice. For natalizumab, which carries the highest risk (9), patients are tested for JCV antibodies using the STRATIFY JCV assay (17, 18). However, the specificity of this test is low because JCV is ubiquitous in the general population (18, 19) and even JCV-negative patients are not free of risk since ~11% will seroconvert and thus be in the higher risk group (20). Confounding the risk decision process for the clinician and patient is natalizumab's high efficacy (21, 22), leading many JCV-positive patients to take it despite the risk of PML. Clearly there is a high unmet need for a more effective PML risk prediction test.

Host genetics were hypothesized to predispose individuals to PML (23) and subsequent case reports and studies further support the association of PML with an underlying immunodeficiency disorder (24–28). Immune dysregulation disorders, previously classified as primary immunodeficiency diseases (PID or PIDD) but now termed inborn errors of immunity, are highly heterogeneous with 344 genes now recognized by the International Union of Immunological Societies (IUIS) (29, 30). To date, mutations in 11 IUIS genes (*BTK, CD40LG, DOCK8, MAGT1, NFKB1, PRKD*C, *RAG1, RMRP, STAT1, STK4*, and *WAS*) have been reported in PML patients, most frequently for *DOCK8* (3 cases) (28, 31) and *STAT1* (4 cases) (26, 32). In addition to IUIS-designated genes, mutations in *BAG3* were reported as a potential cause of PML in an immunocompetent patient based, in part, on the gene's links to JCV (e.g., virus replication is reduced when *BAG3* is over-expressed) (33).

As a side note, a connection between host genetics and severe complications in a small subset of virus-infected patients occurs not only in the context of JCV. Hatchwell (23) cited two other examples, X-linked lymphoproliferative disorder with Epstein-Barr virus and TLR3 deficiency with herpes simplex virus 1, which was originally reported in 2007 (34). Several other examples have been discussed in reviews (35, 36).

Given the high unmet need for PML risk prediction and the preliminary evidence that host genetics may be a key factor (23), we sought to identify PML-associated genetic variants in two cohorts of PML patients (*n* = 70 Discovery cases, *n* = 115 Replication cases). We performed whole exome sequencing (WES) on the 185 PML cases to enable an investigation of all genes, but focused our study on identifying rare variants in a set of 669 immune function genes. Using a variant burden approach, we identified 19 variants in 17 genes (10 are IUIS genes) that are candidates for a PML genetic risk prediction test.

## Methods

### PML Cases Reported in the FDA Adverse Event Reporting System (FAERS)

The current landscape of drugs potentially linked to PML was assessed using publicly available data from the FDA (https://open.fda.gov/data/faers/), accessed on November 2, 2019. Using the search term “progressive multifocal leukoencephalopathy,” data were downloaded for all years (1997 through June 30, 2019), a total of 4,854 cases. The data were filtered on the basis of the patient's underlying condition(s) (Reason for Use) and drug/biologic used (Suspect Product Active Ingredients).

**Figure 1:**
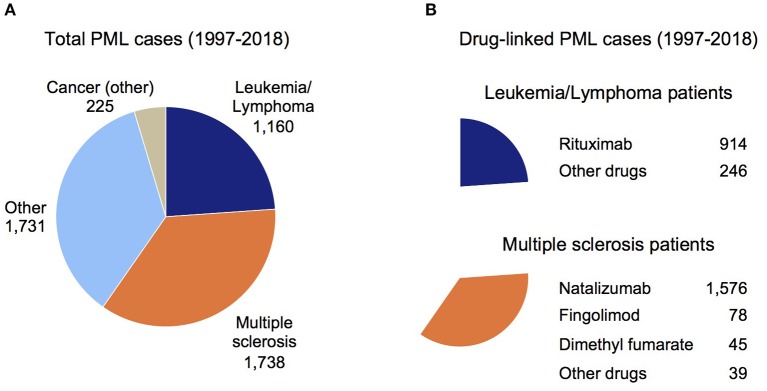
Overview of PML cases in the FDA Adverse Event Reporting System (FAERS). **(A)** Total PML cases reported in the FAERS database sub-grouped by the patient's underlying disease (see Methods). **(B)** Total drug-linked PML cases for the Leukemia/Lymphoma patients (top chart) and MS patients (bottom chart). The largest number of drug-linked PML cases in these disease groups were found for rituximab and natalizumab (Other drugs subgroup contains drugs linked to a small number of PML cases).

For Figure 1A, Reason for Use conditions were binned as Cancer (other), Leukemia/Lymphoma, MS, and Other (cases where the indication was unknown or a variety of disorders such as HIV, rheumatoid arthritis, systemic lupus erythematosus, and transplant patients). For Figure 1B, Suspect Product Active Ingredients were assessed for two subgroups of Figure 1A, Leukemia/Lymphoma (top chart) and MS (bottom chart).

Since PML is a well-established side effect of natalizumab (9, 19) and natalizumab comprises a large number of PML cases in the FAERS data (1,658 of 4,854 cases had natalizumab as a Suspect Active Ingredient), all MS patients with other active ingredients co-listed were assumed to have PML due to natalizumab. For dimethyl fumarate, fingolimod, and other drugs (alemtuzumab, glatiramer acetate, interferon beta, ocrelizumab, and teriflunomide), PML was attributed to these active ingredients only if natalizumab was not co-listed. Figure 1B shows the breakdown of PML cases by drug for the Leukemia/Lymphoma and MS subgroups (Figure 1A).

### PML Cases

Written informed consent was obtained from all PML patients participating in this study under IRB approved protocols from the following institutions: Beth Israel Deaconess Medical Center (PI Koralnik), Icahn School of Medicine at Mount Sinai (BioMe Biobank), NINDS/NIH (PI Major), Paris-Sud (PI Taoufik), Vanderbilt University (BioVU Biobank).

Two PML cohorts were assembled for our study: Discovery (Dis) cohort of 70 PML cases from NINDS/NIH (8 cases) and Paris-Sud (62 cases); Replication (Rep) cohort of 115 PML cases from Paris-Sud (24 cases), Beth Israel Deaconess Medical Center (73 cases), Icahn School of Medicine BioMe Biobank (9 cases), and Nashville Biosciences BioVU Biobank (9 cases). Patient demographics and PML diagnostic criteria (7) are summarized in Table 1. Prior to onset of PML, most patients had a primary disease that we broadly grouped as: blood cancers (including chronic lymphocytic leukemia, Hodgkin lymphoma, non-Hodgkin lymphoma, myeloma, and myelodysplastic syndrome), HIV, MS, or Other (including bone marrow, kidney and liver transplant patients, alcoholic cirrhosis, anaplastic plasmacytoma, aplastic anemia, inflammatory myopathy, colon cancer, liver cancer, lymphopenia, polycythemia vera, sarcoidosis, thymoma with immunodeficiency, or unknown).

**Table 1:** Demographics and diagnostic criteria for the PML cohorts.

	**Discovery (*****n*** **=** **70)**		**Replication (*****n*** **=** **115)**		**Total cases (*****n*** **=** **185)**	
**GENDER**						
Male	44		74		118	
Female	26		41		67	
**ETHNICITY**						
EUR	49		87		136	
AFR	21		28		49	
**PRIMARY DISEASE (EUR, AFR)**						
Blood cancer	4	(4, 0)	18	(18, 0)	22	(22, 0)
HIV	53	(32, 21)	72	(45, 27)	125	(77, 48)
MS	9	(9, 0)	6	(6, 0)	15	(15, 0)
Other	4	(4, 0)	19	(18, 1)	23	(22, 1)
**PML DIAGNOSTIC CRITERIA**						
Compatible clinical features	70		115		185	
Compatible imaging findings	70		115		185	
CSF PCR for JCV is positive	70		94		164	
Brain biopsy	0		17		17	

To confirm the ancestry of each PML case, whole genome sequencing (WGS) data at a read depth of 0.1× was generated on genomic DNA for all 185 cases (Gencove, New York, NY). This low pass sequencing method yields sufficient data (on the basis of SNPs) to ascertain ethnicity and to determine uniqueness of a sample (i.e., we confirmed that all 185 DNA samples were obtained from different, unrelated individuals). To simplify interpretation of the PML patient variants using public databases (see Control Subjects), the Gencove-reported ethnicities were combined to determine the highest percentage ethnicity for either European (EUR) or African (AFR) ancestry as follows: EUR = (EMED + NEEUROPE + NEUROPE + NITALY + NNEUROPE + SCANDINAVIA + SWEUROPE) and AFR = (CAFRICA + EAFRICA + NAFRICA + SAFRICA + WAFRICA). The majority of PML patients could be readily assigned as EUR or AFR ethnicity, but a few individuals were binned according to their closest ethnicity (e.g., 5 patients with predominantly ASHKENAZI and 2 patients with predominantly TURK-IRAN-CAUCASUS ancestry were assigned as EUR).

### Control Subjects

Two publicly available population data resources were used as controls for variant interpretation: Genome Aggregation Database (gnomAD, v 2.1.1) (37) containing 8,128 AFR exomes, 4,359 AFR genomes, 56,885 NFE exomes, 7,718 NFE genomes; 1,000 Genomes Project (TGP) (38) containing 440 AFR exomes and 436 EUR exomes. For the TGP controls, variants for the exome data were called using Google's DeepVariant (DV, v 0.7.0) caller (39).

### Whole Exome Sequencing

WES data were generated for the 185 PML cases using the ACE Exome sequencing service by Personalis (Menlo Park, CA). As was done for the TGP exome data, we used the DV caller (39) for variant calling in the WES data sets. Individual BAM files were generated using Burrows-Wheeler Aligner (BWA, v 0.6.2 for Dis cohort, v 0.7.12 for Rep cohort) (40): aligning reads to the Genome Reference Consortium Human Build 37 (GRCh37), realigned using GATK IndelRealigner (https://software.broadinstitute.org/gatk/; v 3.1 for Dis cohort, v 3.4 for Rep cohort), and variant call format (VCF) files were subsequently generated for each PML sample (Dis or Rep cohort). Variant inclusion criteria were: total (read) depth (DP) ≥ 10 [site depths were calculated directly from the BAM files using SAMtools (41) depth function v 1.9, with constraints for Base Quality > 10 and Mapping Quality > 20], Variant Allele Frequency (VAF) ≥ 0.2 for heterozygous calls, and VAF ≥ 0.8 for homozygous calls (VAF < 0.8 were treated as heterozygous calls). Variants that did not pass these filtering criteria were identified as “NA” (DP < 10 and/or VAF < 0.2) and these were not included in read count analyses for the Dis and Rep cohorts. Annotation of DV-called variants was performed using dbNSFP (v 3.5) (42).

### Immune Function Genes

A list of candidate immune function genes was curated from the following main sources: genome-wide study of rare copy number variants in 70 PML cases (Dis cohort) that impact immune function genes (data not shown), genes from the ClinVar database (43) using search terms “immune deficiency” and “immunodeficiency,” IUIS and other immunodeficiency reviews (29, 30, 44–50), type I interferon pathway genes (51–58), complement pathway genes (59), and JCV or PML linked biology (23, 26, 33, 60–64). The full list of 711 unique genes were cross-checked against genes that were found with DV-called variants in the Dis and/or Rep cohorts. This yielded a set of 669 unique genes (Supplementary Table 1) that were used for variant burden analyses.

### Statistical Analyses

Odds ratios (ORs) and Fisher's Exact Test (2-tail) *p*-values were calculated with a custom perl script (perl module: https://metacpan.org/pod/Text::NSP::Measures::2D::Fisher::twotailed) or R: RStudio Team (2015), RStudio: Integrated Development for R. RStudio, Inc., Boston, MA URL http://www.rstudio.com/. Calculations in **Table 4** were performed based on the method described in Tonk et al. (65).

### Variant Burden Analyses

Variant burden analyses on the Dis and Rep PML cohorts were performed by preparing a set of four files containing heterozygous (het) variants for PML cases compared to gnomAD data (v 2.1.1, both exomes and genomes were used): Non-Finnish Europeans (NFE) for EUR PML cases and African/African American (AFR) for AFR PML cases. Separate files were prepared for all genes (~20,000) and the curated set of 669 immune function genes (Supplementary Table 1): 669-genes Dis, all-genes Dis, 669-genes Rep, all-genes Rep. For each variant found in at least one PML case, a count was performed in order to obtain the frequency of a given variant in the cohort as a whole. This aggregate data was compared to counts for the same variant as reported in gnomAD. For filtering purposes, the variant burden files included functional prediction annotation (e.g., PolyPhen and SIFT), variant frequency in the PML cohorts and gnomAD subjects (i.e., number of het PML cases/total PML cases or number of het gnomAD subjects/total gnomAD subjects), OR, and *p*-value.

Filtering of candidate variants was done on an ethnic-specific basis (EUR or AFR) and with both ethnicities combined (EUR+AFR). Top candidate variants in the 669-genes variant burden files (Dis and Rep) were identified on the basis of the following filtering criteria (Supplementary Table 2): Impact = High or Moderate, gnomAD frequency ≤ 0.05, number of PML cases with a given variant (PML_ALT) was set at ≥1, 2, or 3 (AFR, EUR, EUR+AFR, respectively), OR > 1, and *p* ≤ 0.1. In the pair of all-genes variant burden files, the filtering criteria were: Impact = High or Moderate, gnomAD frequency ≤ 0.05, number of PML cases with a given variant (PML_ALT) was set at ≥1, 3, or 3 (AFR, EUR, EUR+AFR, respectively), OR > 1, and *p*-value was set at ≤ 0.005, 0.05, or 0.01 (AFR, EUR, EUR+AFR, respectively).

Further steps for identifying top candidate variants consisted of: (1) determining the set of filtered variants found in both the Dis and Rep data (Overlap Dis Rep), (2) technical review, and (3) gene biology review. For technical review, a rank of 1 was assigned for variants meeting the following criteria: Overlap Dis Rep variants had an OR > 3 in both the Dis and Rep cohorts, were present on an autosomal chromosome (one chromosome X variant was excluded, which would be impractical to interpret), had maximum read counts in the PML cases (Dis EUR = 49, Dis AFR = 21, Rep EUR = 136, Rep AFR = 28), had >80% coverage in both the NFE and AFR gnomAD subjects (i.e., the variant was reported in >62,000 NFE+AFR, ~80% of maximum 77,090), and had reasonable PML case counts and *p*-values (occasional variants with unlikely *p*-values received extra scrutiny, such as inspection of the BAM images, to exclude false positives based on factors not adequately dealt with by the usual QC filtering criteria—e.g., pseudogenes or complicated insertions/deletions). For gene biology, a rank of 1 was assigned to all variants in the 669-genes files (i.e., their corresponding genes implicitly have strong biology, see Supplementary Table 1) and PubMed was used to find supporting immune dysregulation biology for any all-genes candidates.

A set of 19 variants, reported in Table 2, were considered to be candidates for a PML genetic risk test. The OR and *p*-values were calculated on an ethnic-specific basis for the individual cohorts (Dis or Rep) and for the combined cohorts (Dis plus Rep) using gnomAD subjects as the control data. We note that while there were no homozygous PML cases for the set of 19 variants, 6 of 19 variants had a small number of homozygotes reported in gnomAD and these were included assuming a dominant model (i.e., hets and homs were summed for the calculations). Variants are sorted by descending OR value for the combined cohort data except for *PLCG2* variant 16-81942175-A-G, which is the only variant that survived the filtering and ranking criteria for the EUR+AFR 669-genes analysis. Its OR and *p*-value are reported three ways (EUR, AFR, and EUR+AFR). Finally, less stringent filters were applied in a second set of analyses but only for the set of 17 genes identified in the first set of filters. The second level of filtering required presence in the combined cohorts (Dis plus Rep) of ≥3 EUR cases or ≥2 AFR cases and at least 1 case in both the Dis and Rep cohorts. Only one additional variant was found and added to Table 2, an EUR variant (*IGLL1* 22-23915745-G-A).

**Table 2:** PML cohort variant burden results for 669 immune function genes and all genes analyses.

**Analysis^a^**	**Gene symbol**	**Variant (hg19)**	**Ethnicity**	**gnomAD subjects^b^**	**Discovery**			**Replication**			**Combined**			
					**PML cases**	***p*-value**	**OR**	**PML cases**	***p*-value**	**OR**	**PML cases**	***p*-value**	**OR**	**OR 95% CI**
1	*IGLL1*	22-23915745-G-A	EUR	0/48/64410	2/49	6.77E-04	57.02	1/87	6.40E-02	15.59	3/136	1.77E-04	30.23	5.95–95.84
2	*MDC1*	6-30673359-T-G	EUR	0/347/60562	2/49	3.25E-02	7.38	6/87	1.27E-05	12.85	8/136	1.55E-06	10.85	4.55–22.25
2	*STXBP2*	19-7712287-G-C	EUR	0/311/63507	2/49	2.44E-02	8.65	2/87	6.88E-02	4.78	4/136	4.81E-03	6.16	1.64–16.31
3	*FCN2*	9-137779251-G-A	EUR	2/505/64168	3/49	7.00E-03	8.19	3/87	3.23E-02	4.48	6/136	8.21E-04	5.79	2.08–13.04
2, 3	*MCM5*	22-35806756-G-A	EUR	7/736/64588	3/49	1.90E-02	5.60	4/87	1.85E-02	4.14	7/136	1.10E-03	4.66	1.83–9.93
2	*IGLL1*	22-23915583-T-C	EUR	0/541/64515	2/49	6.41E-02	5.03	3/87	3.74E-02	4.22	5/136	6.14E-03	4.51	1.44–10.87
2	*IFIH1*	2-163136505-C-G	EUR	6/1367/64143	6/49	6.18E-04	6.38	5/87	3.95E-02	2.79	11/136	1.86E-04	4.02	1.95–7.47
2	*PLCG2*	16-81939089-T-C	EUR	3/610/64302	2/49	7.99E-02	4.42	3/87	5.12E-02	3.71	5/136	1.03E-02	3.97	1.26–9.54
4	*PLCG2*	16-81942175-A-G	EUR	2/934/64000	3/49	3.52E-02	4.39	3/87	1.36E-01	2.41	6/136	1.56E-02	3.11	1.12–6.98
5	*LY9*	1-160769595-AG-A	AFR	0/0/12479	1/21	1.68E-03	1826.27	1/28	2.24E-03	1361.40	2/49	1.50E-05	1313.63	48.37–Inf
6	*LIG1*	19-48643270-C-T	AFR	0/10/12484	1/21	1.83E-02	62.11	1/28	2.44E-02	46.02	2/49	9.64E-04	52.87	5.49–260.10
6	*PKHD1*	6-51798908-C-T	AFR	0/17/12485	2/21	4.51E-04	76.87	1/28	3.95E-02	27.12	3/49	6.11E-05	47.68	8.66–173.38
6	*AIRE*	21-45708278-G-A	AFR	0/23/12433	1/21	3.97E-02	26.94	1/28	5.26E-02	19.95	2/49	4.28E-03	22.94	2.55–97.1
6	*GFI1*	1-92946625-G-C	AFR	0/30/12105	1/21	5.24E-02	20.09	1/28	6.92E-02	14.89	2/49	7.31E-03	17.11	1.93–70.83
6	*NQO2*	6-3015818-G-A	AFR	0/54/12484	1/21	8.85E-02	11.50	2/28	6.90E-03	17.69	3/49	1.42E-03	15.00	2.9–49.01
6	*C8B*	1-57409459-C-A	AFR	0/38/12483	1/21	6.35E-02	16.35	1/28	8.38E-02	12.12	2/49	1.06E-02	13.93	1.58–56.58
6	*CFHR2*	1-196918605-A-G	AFR	0/58/12384	1/21	9.53E-02	10.62	2/28	8.01E-03	16.33	3/49	1.76E-03	13.85	2.68–45.01
6	*DNASE1L3*	3-58191230-G-T	AFR	0/44/12483	1/21	7.30E-02	14.12	1/28	9.61E-02	10.46	2/49	1.39E-02	12.02	1.37–48.52
6	*TCIRG1*	11-67818269-G-A	AFR	2/490/12463	4/21	8.60E-03	5.72	3/28	9.76E-02	2.92	7/49	3.09E-03	4.05	1.53–9.17
4	*PLCG2*	16-81942175-A-G	AFR	0/194/12067	4/21	3.36E-04	14.39	1/28	3.66E-01	2.27	5/49	1.21E-03	6.95	2.13–17.76
4	*PLCG2*	16-81942175-A-G	EUR+AFR	2/1128/76067	7/70	8.60E-05	7.37	4/115	9.34E-02	2.39	11/185	1.22E-04	4.19	2.05–7.72

a *Analysis source of the variant: 1, ad-hoc EUR for 669-genes; 2, 669-genes EUR; 3, all-genes EUR; 4, 669-genes EUR+AFR; 5, all-genes AFR; 6, 669-genes AFR*.

b *Allele data are reported as homozygotes/heterozygotes/total subjects (allele number/2)*.

Two more quality checks were performed for the set of 19 variants to determine if there was any bias in the statistical analyses by using the full set of gnomAD data (joint variant calling using a BWA-Picard-GATK pipeline). First, *p*-values and OR were calculated using only the gnomAD exome data (GE) and the gnomAD genome data (GG). This was done for the combined PML cohorts (Dis plus Rep) and, for comparison, the corresponding Table 2 results (GE+GG) were reiterated in Supplementary Table 3. Second, a fourth set of *p*-values and OR were calculated using the DV-called TGP exome data (38) for EUR (436 subjects) and AFR (440 subjects) ancestries to check if there were any major discrepancies between the gnomAD variant calling and DV calling (PML cases and TGP exome subjects). Comparison data (*p*-values and OR) for the four approaches are reported in Supplementary Table 3.

### Functional Impact and Immune Dysfunction Biology for Top Variants

Supplementary Table 4 reports on the functional impact for the top 19 variants (their *p*-values and OR are reported in Table 2) and 17 genes in which they are found (*IGLL1* and *PLCG2* each had 2 variants). In addition to the IUIS gene information reported in Supplementary Table 1, further evidence of immune dysfunction for the genes and/or variants from prior studies is also reported in Supplementary Table 4.

### Diagnostic Yield, Clinical Validity, and Population Impact Analyses

Diagnostic yield (PML cases with a variant divided by total PML cases assessed for the variant) for the top 19 variants are reported in Table 3, individually within the EUR or AFR combined cohorts and for the non-redundant cumulative number of cases (a subset of the PML cases had 2–3 of 19 variants). Table 3 also reports the distribution of the 19 variants by primary disease subgroups (Blood cancer, HIV, MS, and Other) and if they are found in the other ethnicity (EUR variants in AFR cases or AFR variants in EUR cases). Variant IDs in Table 3 reference which variants were included in variant panel calculations presented in Table 4.

**Table 3:** Diagnostic yield and distribution of top 19 PML-associated variants by primary disease.

**Variant ID**	**Gene symbol**	**Variant (hg19)**	**Cases per variant**	**Cum. cases non-redundant**	**Cum. Dx yield**	**Blood cancer (*n* = 22)**	**HIV (*n* = 125)**	**MS (*n* = 15)**	**Other (*n* = 23)**	**Other ethnicity cases^a^**
**COMBINED EUR COHORTS (*****n*** **=** **136)**										
1	*IGLL1*	22-23915745-G-A	3	3	2.2%	0	2	1	0	1 HIV
2	*MDC1*	6-30673359-T-G	8	10	7.4%	0	7	0	1	1 HIV
3	*STXBP2*	19-7712287-G-C	4	14	10.3%	0	1	2	1	0
4	*FCN2*	9-137779251-G-A	6	19	14.0%	1	3	2	0	1 HIV
5	*MCM5*	22-35806756-G-A	7	26	19.1%	0	5	1	1	0
6	*IGLL1*	22-23915583-T-C	5	31	22.8%	4	1	0	0	0
7	*IFIH1*	2-163136505-C-G	11	40	29.4%	1	7	1	2	1 HIV
8	*PLCG2*	16-81939089-T-C	5	44	32.4%	0	3	0	2	0
9	*PLCG2*	16-81942175-A-G	6	45	33.1%	0	3	1	2	5 HIV
**COMBINED AFR COHORTS (*****n*** **=** **49)**										
10	*LY9*	1-160769595-AG-A	2	2	4.1%	0	1	0	1	0
11	*LIG1*	19-48643270-C-T	2	4	8.2%	0	2	0	0	1 HIV
12	*PKHD1*	6-51798908-C-T	3	7	14.3%	0	3	0	0	0
13	*AIRE*	21-45708278-G-A	2	9	18.4%	0	2	0	0	0
14	*GFI1*	1-92946625-G-C	2	11	22.4%	0	2	0	0	1 Blood cancer, 1 HIV
15	*NQO2*	6-3015818-G-A	3	14	28.6%	0	3	0	0	0
16	*C8B*	1-57409459-C-A	2	16	32.7%	0	2	0	0	1 Blood cancer, 1 HIV, 1 MS
17	*CFHR2*	1-196918605-A-G	3	19	38.8%	0	3	0	0	0
18	*DNASE1L3*	3-58191230-G-T	2	20	40.8%	0	2	0	0	0
19	*TCIRG1*	11-67818269-G-A	7	24	49.0%	0	7	0	0	0
9	*PLCG2*	16-81942175-A-G	5	26	53.1%	0	5	0	0	3 HIV, 1 MS, 2 Other

a *Other ethnicity cases lists the number of AFR cases with an EUR variant (1–9) or the number of EUR cases with an AFR variant (9, 10–19)*.

**Table 4:** Modeling clinical validity and population impact of variants for a natalizumab PML genetic risk test^a^.

**Measure^b^**	**EUR all (*n* = 136)**	**EUR non-MS (*n* = 121)**	**EUR MS (*n* = 15)**	**EUR MS (*n* = 15)**	**AFR all (*n* = 49)**
Variant IDs^c^	1–9	1–9	1–9	1, 3, 4	10–19, 9
Presence in PML cases	33.1%	30.6%	53.3%	33.3%	53.1%
Presence in gnomAD subjects	8.5%	8.5%	8.5%	1.4%	7.7%
Adverse event frequency^d^	1.3%	1.3%	1.3%	1.3%	1.3%
*p*-value	6.23E-16	3.30E-12	1.01E-05	1.23E-06	1.71E-16
OR [95% CI]	5.33 [3.64–7.70]	4.75 [3.13–7.07]	12.31 [3.90–39.90]	36.46 [9.75–117.60]	13.46 [7.35–24.79]
Sensitivity	32.2%	29.8%	51.5%	27.2%	51.0%
Specificity	91.8%	91.8%	92.1%	99.0%	92.8%
PPV	4.9%	4.6%	7.9%	26.1%	8.6%
NPV	99.0%	99.0%	99.3%	99.0%	99.3%
PAF	25.9%	23.3%	47.0%	26.2%	46.9%
NNT	25	28	14	4	13
NNG	297	330	164	294	164
NNT/NNG	0.08494	0.08494	0.08494	0.01353	0.07745
MS cases excluded from therapy^e^	4,672	4,672	4,672	744	4,260
PML cases prevented^f^	108	97	196	109	196

a *Data are reported on an ethnic-specific basis for the combined PML cohorts (Dis and Rep); subsets of the EUR cohort (non-MS, MS all 9 variants, MS top 3 variants) are also reported*.

b *Measure abbreviations reported in Tonk et al. (65): PPV, positive predictive value; NPV, negative predictive; PAF, population attributable fraction; NNT, number needed to treat; NNG, number needed to genotype*.

c *See Table 3 for variants IDs*.

d *Natalizumab manufacturer's maximal reported incidence of PML (13/1,000), accessed on November 9, 2019: www.tysabrihcp.com/en_us/home/efficacy-safety/pml-risk.html*.

e *Estimated number of MS cases excluded from therapy = NNT/NNG × 55,000 JCV-positive MS patients (19)*.

f *Estimated number of PML cases prevented = PAF × 418 JCV-positive PML cases (19)*.

**Figure 2:**
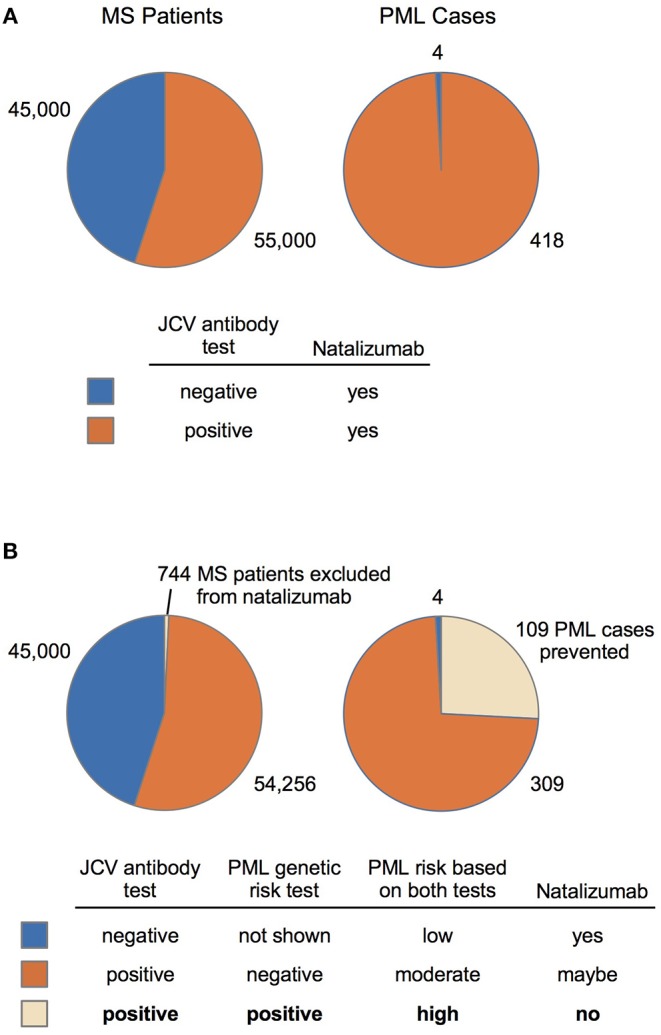
MS-Natalizumab patient population (2014–2016) and predicted performance of a PML genetic risk test. **(A)** Number of MS patients (JCV-negative vs. JCV-positive) on natalizumab and the subset that developed PML are based on a summary by Schwab et al. (19). **(B)** Predicted impact of a PML genetic risk test is based on the population impact data reported in Table 4 (EUR MS, variants 1, 3, and 4). A suggested diagnostic decision process (bottom panel) is depicted for patients that test positive and/or negative on the JCV antibody test (currently used in natalizumab treatment decisions) and the proposed PML genetic risk test.

Clinical validity and population impact (65) of the top 19 variants for the PML-linked biologic drug natalizumab are reported in Table 4. The adverse event frequency was set at 1.3% based on the manufacturer's current maximal estimate of PML incidence (www.tysabrihcp.com/en_us/home/efficacy-safety/pml-risk.html), which is comparable to 1.28% reported for the STRATA study (66). Using the real world natalizumab data summarized in Schwab et al. (19), the predicted performance of a PML genetic risk test are depicted in Figure 2 using the top three variants (1, 3, and 4) for the EUR MS subgroup of PML cases (*n* = 15).

## Results

Given the high unmet need for drug-linked PML risk prediction and preliminary data that PML patients have an underlying immunodeficiency disorder, we sought to identify germline variants in PML cases for development of a PML genetic risk test. Two PML cohorts were assembled for our study and WES data were generated for the total set of 185 cases. The primary analyses focused on a set of 669 immune function genes but all genes were also investigated with a more stringent set of filters. To develop a simple genetic test, our goal was to identify rare variants that were found in both PML cohorts and at a higher frequency than found in the general population.

### Highest Number of Drug-Linked PML Cases Are Reported for Natalizumab and Rituximab

To understand the current landscape for drug-linked PML and confirm the need for a PML genetic risk test, we searched for PML cases in the publicly available FAERS database (1997 through June 2019). While duplicative reports are a caveat to its use, this is offset by the substantial underreporting of SAE case reports in FAERS (67). One other limitation is that likely not all FAERS-reported PML cases have received a formal diagnosis of definite PML, but will instead be probable or possible cases (7). Figure 1A shows the total set of 4,854 PML patients sub-grouped by their primary disease: Cancer (other), Leukemia/Lymphoma, MS, or Other (see Methods). Figure 1B shows rituximab is the main drug reported for PML patients with Leukemia/Lymphoma (top panel) and for the MS subgroup it is natalizumab (bottom panel).

The incidence of PML in patients on rituximab has been estimated at 1 in 30,000 according to a 2016 review (9). In a 2019 review (14), incidence was estimated at 1 in ~13,000 (7–8 per 100,000) but could be even more frequent in some patient groups (e.g., two Veteran's Administration hospital studies were cited, 7/8,895 in non-HIV non-Hodgkin's lymphoma patients and 4/2,425 non-HIV chronic lymphocytic leukemia patients). Rituximab is now being used to treat a broader range of diseases (e.g., rheumatoid arthritis, granulomatosis with polyangiitis, and microscopic polyangiitis), including off-label for MS, so the total number of patients at risk of developing PML while on rituximab is growing (13, 14).

Association of PML with natalizumab therapy is well-established, most notably in MS patients for which the drug was originally approved (3, 16, 19, 68). In MS patients (Figure 1B), an appreciable number of PML cases were reported for fingolimod and dimethyl fumarate, but natalizumab accounts for 91% of FAERS-reported PML patients (1,576 cases). The manufacturer of natalizumab reported a decline in PML incidence since the introduction of the JCV antibody test (69), but other analyses indicate it may be underestimated (19, 70). Compared to other drugs, PML risk for patients on natalizumab is at least ~20 times higher (e.g., 1/100–1/1,000 for natalizumab vs. 1/18,000 for fingolimod) (9). The FAERS data for MS patients on natalizumab over the past 5 years (2014–2018) that developed PML are: 178, 447, 155, 221, 160. Even with exclusion of the potential outlier 2015 report of 447 cases, this is an average of 179 PML cases per year and is further evidence that PML continues to be a significant issue despite use of the JCV antibody test.

### Assembly of Two Large PML Cohorts to Identify Germline Genetic Risk Variants

To maximize our ability to discover germline genetic risk variants of PML, we assembled two large cohorts of PML cases (Table 1). We believe this is the largest study, to date, for investigation of host genetics that predispose individuals to developing PML. The Dis cohort is smaller than the Rep cohort, but the demographics are comparable (Dis vs. Rep): greater number of males (63 vs. 64%), greater number of EUR cases (70 vs. 76%), and HIV is the largest primary disease subgroup (76 vs. 63%). The distribution of the total number of PML cases (Dis plus Rep) in our study approximates the percentages reported by the PML Consortium (15), wherein the largest number of cases occur in HIV patients (~80%), followed by hematological malignancies (~10%), other conditions (<10%), and MS patients on natalizumab (<5%).

PML diagnostic criteria used for the cohorts were essentially those defined in a consensus statement paper (7). One “gold standard” criterion for diagnosing PML is a positive CSF test for JCV via a PCR assay, which was reported for 89% of our PML patients. Compatible clinical and imaging features were found for all of our patients (Table 1), whereas brain biopsy was available for only 9% of patients.

### Evidence of Underlying Immunodeficiency Disorders in PML Cases

Our study design is based on the hypothesis that PML occurs in patients with an underlying immunodeficiency disorder (23). Preliminary evidence for this premise is supported by reports of PML cases with mutations in IUIS immunodeficiency disorder genes (29, 30), such as *DOCK8* and *STAT1* plus others (23–28, 31, 32). Therefore, we curated a set of 669 immune function genes (Supplementary Table 1) of which 337 (50%) are reported to cause inborn errors of immunity (29, 30) and the rest are from a variety of sources (see Methods). This 669-genes list was used for our main genetic analyses of the PML patient WES data in order to enhance the discovery of PML-associated risk variants (i.e., based on biology and statistics).

### Rare, Deleterious Variants in Immune Function Genes Identified in Both PML Cohorts

Using a variant burden approach (see Methods), we identified 19 variants in 17 genes (Table 2) that are candidates for a PML risk test. Most of these variants (17 of 19) were found by searching within the 669-genes data, while the remaining 2 variants (*FCN2* and *LY9* genes) were discovered using the entire WES data set with alternate filtering criteria (see Methods). Supplementary Table 2 summarizes the filtering criteria that were applied to the variant burden files. In the 669-genes analyses, filtering reduced the variants considered as candidates in the Dis and Rep cohorts to <100 in the EUR and EUR+AFR analyses and <400 in the AFR analysis. In the all-genes analyses, filtering yielded <600 candidate variants in the EUR and EUR+AFR analyses and <1,700 in the AFR analysis.

An important aspect of our analyses is that our Dis and Rep cohort sizes were underpowered to apply discovery and replication methods typically used in GWAS. Because PML is so rare, whether due to an immunosuppressed condition (e.g., HIV or hematological disorders) or drug-related (e.g., natalizumab or rituximab), it is not possible to assemble patient cohorts in the thousands even via a worldwide consortium. Instead, we assessed the set of filtered variants that overlapped between the Dis and Rep cohorts (Supplementary Table 2) and then applied technical and biological ranking criteria (see Methods) to yield the final set of candidate PML risk variants.

Variants statistics (*p*-values and OR) using gnomAD subjects as controls are presented in Table 2 on an ethnic-specific level (EUR or AFR) for the Dis and Rep cohorts and for the Combined cohort (Dis plus Rep). We note that others have successfully used large population-based cohorts to investigate rare disease (71). To ensure there was minimal bias in using the full set of gnomAD data (genomes and exomes) as controls, we also performed the statistical calculations separately for gnomAD genomes, gnomAD exomes, and TGP exomes (Supplementary Table 3). The three additional sets of analyses were compared to the Table 2 statistics but no appreciable differences in direction or magnitude of effect were found.

In Supplementary Table 4, we report on the allele frequency (gnomAD) and functional impact for the 19 variants reported in Table 2 along with additional biological support for the corresponding genes from the published literature. For functional prediction variant filtering, we restricted our analyses to variants with a high or moderate impact but did not require that a variant was predicted to be deleterious as results can vary depending on the algorithm used (72). Despite the caveats in variant prediction, 15 of 19 variants were predicted to be deleterious by one or more prediction algorithms (Supplementary Table 4).

All 19 variants, whether found with EUR or AFR filtering methods, had a gnomAD allele frequency of <0.02 and 17 of 19 were < ~0.008 (EUR and AFR allele frequencies are reported in Supplementary Table 4). This is likely a consequence of applying a variant frequency filter of ≤ 0.05 for gnomAD subjects (i.e., twice the allele frequency) in both the EUR and AFR ethnicities prior to filtering on the number of PML cases on an ethnic-specific basis (Supplementary Table 2). Rare variants typically have a higher effect size, a key metric for a predictive test (65), which is why we implemented this in our filtering strategy.

Finally, Supplementary Table 4 summarizes additional immune function biology for the 17 genes in which the top 19 variants were found. We note that our 669-genes immune function list (Supplementary Table 1) is comprised of 337 IUIS genes (29, 30) and 10 of 17 genes found in our study are IUIS genes. Supporting immune function biology is cited for the remaining genes, including the two that were found in the all-genes analyses (*FCN2* and *LY9*).

### PML-Linked Variants Have Cumulative Diagnostic Yields of 30–50% and Are Distributed Across Primary Diseases

Using the top 19 variants in Table 2, we constructed ethnic-specific variant panels (Table 3) as prototypes for a PML risk test that could use cost-effective genotyping methodologies. Variants were ordered (Variant ID in Table 3) by descending OR value (Table 2) within the EUR and AFR PML patient groups. The number of PML cases with each variant is reported on an ethnic-specific basis and in the other ethnicity. For example, Table 3 reports that the variant *IGLL1* 22-23915745-G-A was found in 3 EUR cases (Cases per variant) and 1 AFR case (Other ethnicity cases). Also reported in Table 3 is the non-redundant cumulative number of PML cases with one or more variants comprising the EUR (9 variants, Variant IDs 1–9) or AFR (11 variants, Variant IDs 9–19) panel. The cumulative diagnostic yield was 33.1% for the EUR PML cases (45/136) and 53.1% for the AFR PML cases (26/49).

The distribution of the top 19 variants across the four primary disease subgroups is also reported in Table 3. This analysis was not informative in the AFR subgroup since 48 of 49 AFR PML cases had HIV as their primary disease. In the EUR subgroup, however, all 9 EUR variants in the panel were found in two or more primary diseases and 5 EUR variants were found in three or more primary diseases. The *IFIH1* variant (2-163136505-C-G) was found in EUR PML cases for all four primary disease groups (Blood Cancer, HIV, MS, and Other) plus one AFR case (HIV). Notably, even in the smallest primary disease group of 15 MS cases, 6 of 9 variants were found. Overall, these findings support the candidacy of these variants as predictors of PML risk, rather than causing or contributing to the underlying (primary) disease that each PML case has.

### PML Cases Can Be Prevented With a Germline Genetic Risk Test

Since natalizumab is the therapeutic agent that carries the highest PML risk (9), we investigated the potential impact of our variants in predicting PML risk. Clinical validity and population impact parameters are reported in Table 4 for the EUR (*n* = 136) and AFR (*n* = 49) combined cohorts (Dis plus Rep). Calculations were performed based on Tonk et al. (65), assuming a PML adverse event frequency for natalizumab of 1.3% (see Methods). To model the PML risk test performance in MS patients on natalizumab (see below), we used the JCV-negative and JCV-positive subgroups reported by Schwab et al. (19) (Figure 2) for determining the number of MS cases that would be excluded from natalizumab therapy and the number of PML cases that could be prevented.

Despite only requiring a small number of variants to be genotyped (Table 3, EUR Variant IDs 1-9), the EUR panel had a reasonable sensitivity of 32.2% and specificity was ~92% (Table 4). Since the primary patient group exposed to natalizumab is MS, we further evaluated the EUR panel tests in MS (*n* = 15) and non-MS (*n* = 121) PML cases separately. The non-MS subgroup test parameters were comparable to the EUR all group, but interestingly the EUR variant panel showed higher sensitivity (51.5%) in the MS subgroup. This is an encouraging result as potentially even more cases of PML can be prevented if the sensitivity remains high when further MS PML cases are tested for validation of the 9-variant test panel.

We also modeled an EUR panel test containing the top 3 variants (Table 3, EUR Variant IDs 1, 3, and 4) found in the MS subgroup. While this decreased the sensitivity (51.5% for 9 variants vs. 27.2% for 3 variants), specificity increased from 92 to 99%, which would reduce the number of patients excluded from natalizumab therapy. In the example data (19) reported in Table 4, if 55,000 JCV-positive MS patients were tested with the 9-variant panel, 4,672 patients (8.5%) would be excluded, whereas only 744 patients (1.4%) would be excluded with 3-variant panel. Furthermore, of the 418 PML cases reported for the example data, the 9-variant and 3-variant panels would have prevented 196 (PAF = 47.0%) or 109 (PAF = 26.2%) cases of PML, respectively.

The clinical impact that our 3-variant PML genetic risk test would have in the MS patient community considering natalizumab therapy is modeled in Figure 2. The scenario described by Schwab et al. (19) for 100,000 MS patients is depicted in Figure 2A, wherein the low specificity JCV antibody test (45% estimate in Table 2 of Schwab et al.) means 45,000 patients will be JCV-negative and 55,000 patients will be JCV-positive. The number of PML cases in the MS population is also depicted (Figure 2A, right chart), which predominantly occurs in the JCV-positive patient subgroup (418 cases vs. only 4 in JCV-negative patients). Despite the substantially higher risk of PML in JCV-positive patients, natalizumab continues to be a popular MS therapy because it is highly effective (73).

Figure 2B shows that adding our 3-variant PML risk test (Table 4) to the clinical decision process would exclude only 744 patients from natalizumab treatment but would prevent 109 PML cases in the subset of patients that are JCV-positive. Based on a 76% survival rate for natalizumab-associated PML (74), this would also mean that 26 deaths could have been prevented. Using the FAERS data (Figure 1) calculated average of 179 natalizumab-associated PML cases per year (4-year average excluding high outlier year 2015), on an annual basis our 3-variant or 9-variant PML risk test panels would prevent 47–84 PML cases and 11–20 deaths. Figure 2B (bottom panel) also shows how we envision our PML genetic risk test could be implemented in the clinical setting, wherein patients testing positive for both the JCV antibody test and the genetic risk test would be strongly advised not to take natalizumab.

## Discussion

We describe, for the first time, a set of 19 germline genetic variants (Table 2) identified in PML cases (i.e., host genetics) that are strong candidates for development of a PML risk test (Table 4, Figure 2). With increased clinical use of highly effective disease-modifying therapies in a variety of disorders, there continues to be a substantial number of new PML cases reported each year in FAERS (Figure 1), which experts believe captures only a fraction of drug-linked SAEs (67, 75, 76). The only PML risk test currently in use is the JCV antibody test for patients considering natalizumab treatment. However, it has low specificity so that while ~40% of patients test negative (i.e., have lower risk of developing PML), at least 60% of patients test positive. In other words, only a small subset of JCV-positive patients truly has a high risk of developing PML (i.e., most JCV-positive patients have low PML risk and could benefit from natalizumab therapy). It also requires periodic testing to monitor if JCV-negative patients seroconvert and/or if the JCV index has exceeded the upper index value of 1.5 (18, 77). Other non-genetic biomarker tests have been investigated, such as CD62L/L-selectin and lipid-specific immunoglobulin M bands in patients on natalizumab (19, 78), but none have been routinely implemented in the clinical setting. Therefore, there is a high unmet need for better risk stratification tools and our PML variants could be implemented in a simple, inexpensive genotyping test. Pending further validation studies, we estimate that at least a quarter to as many as half of PML cases could be prevented (Table 4, PAF values of 26.2–47.0%) in MS patients on natalizumab.

A 2010 study of 152 PML cases attempted to identify host risk variants in *HLA* loci but the results were inconclusive (79). Based on a small number of PML case reports, Hatchwell (23) hypothesized that PML patients have a genetic predisposition as a third major risk factor, in addition to JCV infection and immunosuppression (due to disease and/or immunosuppressive therapy). Later case reports and small studies reporting on PML patients with immunodeficiency disorder mutations strongly supports underlying genetics as a risk factor (24–28). In our WES data on 185 PML cases, we also observed rare, deleterious variants (data not shown) in known immunodeficiency disorder genes (29, 30) but our goal for this study was to identify variants occurring in multiple PML cases that could be used in a PML genotyping risk test. All of our candidate PML risk variants (Table 2) had *p* < 0.02 (uncorrected) in the combined cohorts (Dis plus Rep) and moderate to high effect size (OR values of 3.11–1313.63). Supporting biology (10/17 genes are IUIS immunodeficiency disorder genes) and *in silico* functional prediction (15/19 variants are predicted to be damaging/deleterious by at least one method) provide additional support for their candidacy as PML risk prediction variants (Supplementary Table 4).

An important finding in our study is that all 9 variants found in our EUR PML cases were distributed across two or more disease subgroups (Table 3). While we could not assess this in our AFR PML cases (48 of 49 were HIV PML cases), the dispersal of the same variants in the EUR subgroups suggests that PML genetic risk is independent of the primary disease and/or immunosuppressant that led to the patient's development of PML. In other words, our results indicate that the germline risk factors for PML apply to all subgroups, irrespective of underlying disease, which is consistent with the commonalities observed for clinical and diagnostic features (e.g., motor weakness and cognitive changes and similar brain radiological patterns) described in clinical studies of PML (5, 80–82). Therefore, if validated in additional PML cases, our variants could be used in a risk test applicable in multiple clinical settings: HIV-positive patients to ensure compliance with combination antiretroviral therapy (cART), hematological malignancy patients (such as those being considered for rituximab vs. transplant therapy), MS patients under consideration for disease-modifying therapies (natalizumab, fingolimod, dimethyl fumarate, etc.), and others. Patients testing positive with the JCV antibody test and PML genetic risk test, regardless of underlying primary disease or immunosuppressive therapy being used to treat their condition, would be considered very high risk and therefore benefit from closer clinical scrutiny (e.g., more frequent MRI screenings to detect PML lesions before onset of symptoms).

Presently, among the immunosuppressant therapies with PML risk warnings (e.g., FDA warnings and precautions or black box warnings), natalizumab carries the highest risk (1 in 239 patients) (68, 69). Our clinical validity and population impact results (Table 4, Figure 2), modeled for natalizumab with an adverse event frequency of 1.3% (maximal risk reported by the manufacturer, see Methods), show that at least a quarter of PML cases could be prevented. Natalizumab is a highly effective treatment for many MS patients and some would benefit from its use as a first line therapy, such as those with aggressive or early onset forms of the disease (21, 83–85). Furthermore, an MRI surveillance study suggests that therapy duration is potentially not a very effective risk stratification factor (8). Our PML risk test, if administered along with the JCV antibody test, could enable clinicians and patients to more confidently use natalizumab as a first line therapy and for a longer duration in order to ward off disability. Risk stratification guidelines for administration of a JCV antibody test and a genetic risk test are proposed in Table 5.

**Table 5:** Suggested PML risk stratification guidelines if both the JCV antibody and genetic risk tests are implemented.

**JCV antibody test**	**Genetic test**	**Patient PML risk**
Negative	Negative	Low
Negative	Positive	Moderate
Positive	Negative	Moderate
Positive	Positive	High

Limitations of our study include limited (AFR) or no sampling of non-EUR ancestry PML cases and a small number of MS PML cases (15/185 PML cases). In the combined set of 185 PML cases (Table 1), 74% were EUR ancestry and 26% were AFR ancestry. Under-representation of AFR PML cases is concerning, especially with regard to MS since AFR ethnicity cases are reported to have a more progressive form of the disease and there is a higher incidence of MS in the US for African ancestry patients (86–88). Our approach for identifying PML risk variants should also be investigated in other ancestries (e.g., East Asian, South Asian, and Latino). We think some of the most near-term beneficiaries of a PML genetic risk test are MS patients considering natalizumab treatment. Our study included only 15 cases with MS but the clinical validity and population impact data of our PML risk variants in the MS subgroup are encouraging (Table 4, Figure 2). We are actively recruiting additional PML cases of any ancestry, particularly those with MS as their primary disease, for a follow up study.

## Conclusion

Our discovery of 19 candidate PML risk variants in a large cohort of 185 PML cases, if validated and implemented in a genotyping test, would be a significant advance over existing PML risk stratification practices (e.g., JCV antibody test and assessment of prior immunosuppressant use). For the first time, a proportion of very high risk patients—those testing positive for both the JCV antibody and genetic biomarker risk tests—could be advised to avoid higher PML risk immunosuppressant drugs (e.g., natalizumab and rituximab) and monitored more closely for neurological signs of PML. Whereas, low risk patients (JCV-negative, genetic-negative) may benefit from earlier and longer duration treatment with high efficacy therapies, such as natalizumab in MS patients. We predict that clinical adoption of a PML risk genetic test will result in a substantially greater reduction in new drug-linked PML cases compared to existing risk stratification tests/methods.

## Data Availability Statement

The raw data supporting the conclusions of this article will be made available by the authors, without undue reservation, to any qualified researcher. Requests to access the datasets should be directed to Eli Hatchwell (elihatchwell@populationbio.com).

## Ethics Statement

The studies involving human participants were reviewed and approved by Beth Israel Deaconess Medical Center (PI Koralnik), Icahn School of Medicine at Mount Sinai (BioMe Biobank), NINDS/NIH (PI Major), Paris-Sud (PI Taoufik), and Vanderbilt University (BioVU Biobank). The patients/participants provided their written informed consent to participate in this study.

## Author Contributions

PE, YT, and EH: conception and design of the study. IK, BH, EM, JG, BS, HH-C, and YT: provision of study materials and patients. PE, CB, TR, CC, and EH: data analysis and interpretation. PE and EH: wrote the manuscript. All authors: revised/approved the manuscript.

### Conflict of Interest

CB and CC are employees of Emerald Lake Safety LLC. EH (UK) and PE (USA) are employees of Population Bio, Inc. The remaining authors declare that the research was conducted in the absence of any commercial or financial relationships that could be construed as a potential conflict of interest.
