# Supplementary material for: Germline Genetic Risk Variants for Progressive Multifocal Leukoencephalopathy
Source: Front Neurol. 2020 Mar 17;11:186. doi: 10.3389/fneur.2020.00186 (PMC7094807; doi:10.3389/fneur.2020.00186)
Supplement: Supplementary file 1 [file Data_Sheet_2.pdf]

Supplementary Material

1 Supplementary Tables

SUPPLEMENTARY TABLE 1 | Candidate list of 669 immune function genes.

SUPPLEMENTARY TABLE 2 | Variant burden filtering criteria and results for 669 immune function genes or all genes.

SUPPLEMENTARY TABLE 3 | Comparison of statistical analyses for the combined PML cohorts using four control data sets.

SUPPLEMENTARY TABLE 4 | Functional impact of PML-associated variant burden variants and immune dysfunction evidence.

SUPPLEMENTARY TABLE 1 | Candidate list of 669 immune function genes*

Gene symbol	IUIS table	Gene symbol	IUIS table	Gene symbol	IUIS table	Gene symbol	IUIS table
ACADM	other	*CXCL9*	other	*LARP4B*	other	*RIPK3*	other
ACD	other	*CXCR1*	other	*LAT*	1	*RLTPR*	4
ACKR1	other	*CXCR3*	other	*LCK*	1	*RNASE3*	other
ACP5	7	*CXCR4*	6	*LCP2*	other	*RNASEH2A*	7
ACTB	5	*CXorf40A*	other	*LIG1*	2	*RNASEH2B*	7
ACTN4	other	*CYBA*	5	*LIG4*	1	*RNASEH2C*	7
ADA	1	*CYBB*	5, 6	*LPIN2*	7	*RNASEL*	other
ADA2	7	*CYP2S1*	other	*LRBA*	4	*RNF125*	other
ADAM17	7	*DCLRE1B*	2	*LRRK2*	other	*RNF168*	2
ADAR	7	*DCLRE1C*	1	*LYST*	4	*RNF31*	2
ADARB1	other	*DDX1*	other	*MAGEA9B*	other	*RORC*	6
ADGRL2	other	*DDX58*	7	*MAGT1*	4	*RPSA*	6
ADK	other	*DHX58*	other	*MALL*	other	*RPTOR*	other
AGBL4	other	*DKC1*	2	*MALT1*	1	*RSAD2*	other
AICDA	3	*DNAJC21*	5	*MAP3K14*	1	*RTEL1*	2
AIRE	4, 9	*DNASE1L3*	other	*MAP3K2*	other	*SALL2*	other
AK2	1	*DNASE2*	other	*MAPK1*	other	*SAMD9*	2
ALG12	other	*DNER*	other	*MAPK3*	other	*SAMD9L*	2
ALPL	other	*DNMT3B*	2	*MAPK9*	other	*SAMHD1*	7
ANP32B	other	*DOCK2*	1	*MASP2*	8	*SBDS*	5
AP1S3	7	*DOCK8*	1	*MAVS*	other	*SEMA3E*	2
AP3B1	4	*DSC1*	other	*MB21D1*	other	*SERPINA1*	other
AP3B2	other	*DUSP16*	other	*MBL2*	other	*SERPINB2*	other
AP3D1	4	*EBF1*	other	*MCEE*	other	*SERPINB4*	other
APOBEC3A	other	*EDIL3*	other	*MCM4*	2	*SERPINB6*	other
APOBEC3B	other	*EEA1*	other	*MCM5*	other	*SERPING1*	8, 9
APOL1	6	*EGF*	other	*MDC1*	other	*SH2D1A*	4
ARHGEF7	other	*EGR1*	other	*MECP2*	other	*SH3BP2*	7
ARPC1B	2	*EHF*	other	*MEF2C*	other	*SHARPIN*	other
ASH1L	other	*ELANE*	5	*MEFV*	7	*SKIV2L*	other
ASTN2	other	*EMB*	other	*MEX3C*	other	*SLC17A5*	other
ATG12	other	*EPG5*	2	*MFN1*	other	*SLC29A3*	7
ATG16L1	other	*ERCC6L2*	2	*MFN2*	other	*SLC35C1*	5
ATG5	other	*ETF1*	other	*MGAT5*	other	*SLC37A4*	5
ATG7	other	*ETV6*	other	*MKL1*	5	*SLC3A2*	other
ATG9A	other	*EXTL3*	2	*MLH1*	other	*SLC46A1*	2

ATL2	other	*F9*	other	*MMP9*	other	*SLC7A7*	other
ATM	2	*FAAP24*	4	*MOGS*	3	*SLC8A1*	other
ATP6AP1	3	*FADD*	4	*MON1A*	other	*SLC9A1*	other
ATR	other	*FAS*	4, 9	*MON1B*	other	*SMAD2*	other
AUH	other	*FASLG*	4	*MRE11A*	other	*SMAD3*	other
B2M	1	*FAT4*	2	*MS4A1*	3	*SMAD4*	other
BACH1	other	*FCER2*	other	*MSH2*	other	*SMARCAL1*	2
BACH2	4	*FCGR2A*	other	*MSH5*	other	*SMARCD2*	5
BAG3	other	*FCGR3A*	6	*MSH6*	3	*SMC3*	other
BCL10	1	*FCN3*	8	*MSN*	1	*SMURF2*	other
BCL11B	1	*FERMT3*	5	*MTHFD1*	2	*SNAP29*	other
BCL2	other	*FEZ1*	other	*MVK*	7	*SNCA*	other
BDKRB2	other	*FHL2*	other	*MX1*	other	*SNHG3*	other
BLK	other	*FIS1*	other	*MX2*	other	*SNX10*	6
BLM	2	*FOS*	other	*MYD88*	6	*SNX5*	other
BLNK	3	*FOXH1*	other	*MYSM1*	2	*SOCS2*	other
BLOC1S6	other	*FOXN1*	2	*NBAS*	6	*SP110*	2
BMPR2	other	*FOXP3*	4	*NBN*	2	*SP140*	other
BRD4	other	*FPR1*	5	*NCF1*	5	*SPINK5*	2
BTK	3	*FPR2*	other	*NCF2*	5	*SQSTM1*	other
BTLA	other	*FPR3*	other	*NCF4*	5	*SRP54*	other
C11orf65	other	*FUK*	other	*NCSTN*	6	*ST8SIA5*	other
C1QA	8	*G6PC3*	5	*NFAT5*	4	*STAT1*	6
C1QB	8	*G6PD*	5	*NFIC*	other	*STAT2*	6
C1QC	8	*GATA2*	5	*NFIL3*	other	*STAT3*	2, 4, 9
C1R	8	*GDA*	other	*NFKB1*	3	*STAT5B*	2, 9
C1S	8	*GDPD4*	other	*NFKB2*	3	*STIM1*	2
C2	8	*GFI1*	5	*NFKBIA*	2	*STIM2*	other
C3	8	*GINS1*	2	*NHEJ1*	1	*STK3*	other
C4A	8	*GOLGB1*	other	*NHP2*	2	*STK4*	1
C4B	8	*GPATCH2*	other	*NLRC4*	7	*STN1*	2
C5	8	*GPC5*	other	*NLRP1*	7	*STX11*	4
C5AR1	other	*GPRC5A*	other	*NLRP12*	7	*STXBP2*	4
C6	8	*GRAP2*	other	*NLRP2*	other	*SYNCRIP*	other
C7	8	*GRIA3*	other	*NLRP3*	7, 9	*TBXT*	other
C8A	8	*GTPBP4*	other	*NLRX1*	other	*TAP1*	1
C8B	8	*HAX1*	5	*NOD1*	other	*TAP2*	1
C8G	8	*HCN1*	other	*NOD2*	7	*TAPBP*	1
C9	8	*HELLS*	2	*NOP10*	2	*TAZ*	5

CAD	other	*HERC5*	other	*NQO2*	other	*TBC1D15*	other
CAMLG	other	*HERC6*	other	*NRAS*	9	*TBC1D16*	other
CAPZB	other	*HEXA*	other	*NRIP1*	other	*TBC1D17*	other
CARD11	1, 3	*HIVEP1*	other	*NSMCE3*	2	*TBK1*	6
CARD14	7	*HIVEP2*	other	*OAS1*	other	*TBX1*	2
CARD9	6	*HIVEP3*	other	*OAS2*	other	*TCF3*	3
CASP10	4	*HK2*	other	*OAS3*	other	*TCIRG1*	6
CASP8	4	*HMGB1*	other	*OASL*	other	*TCN2*	2
CAV1	other	*HMOX1*	6	*ORAI1*	2	*TEK*	other
CCBE1	2	*HNRNPLL*	other	*ORC4*	other	*TERT*	2
CCDC22	other	*HP*	other	*OSTM1*	6	*TFPI*	other
CCL11	other	*HPCAL1*	other	*OTULIN*	7	*TFRC*	1
CCL2	other	*HPR*	other	*OVOL2*	other	*THBD*	8
CCL5	other	*HTR2A*	other	*PARN*	2	*THBS1*	other
CCR2	other	*HYOU1*	5	*PCCA*	other	*TICAM1*	6
CCR5	other	*ICAM1*	other	*PCCB*	other	*TINF2*	2
CCZ1	other	*ICOS*	1	*PDCD1*	other	*TIRAP*	6
CD180	other	*ICOSLG*	other	*PDCD1LG2*	other	*TLR3*	6
CD19	3	*IDI1*	other	*PDE3B*	other	*TLR4*	other
CD209	other	*IDI2*	other	*PDGFRA*	other	*TMC6*	6
CD22	other	*IDO2*	other	*PDSS2*	other	*TMC8*	6
CD247	1	*IFI35*	other	*PEPD*	4	*TMEM173*	7
CD27	4	*IFIH1*	6, 7	*PGM3*	2	*TNF*	other
CD274	other	*IFIT1*	other	*PHACTR4*	other	*TNFAIP3*	7
CD276	other	*IFIT2*	other	*PIAS1*	other	*TNFRSF10A*	other
CD300LF	other	*IFIT3*	other	*PIAS2*	other	*TNFRSF11A*	6
CD34	other	*IFNAR1*	other	*PIK3CD*	3	*TNFRSF11B*	other
CD36	other	*IFNAR2*	6	*PIK3R1*	3	*TNFRSF13B*	3
CD37	other	*IFNG*	9	*PINK1*	other	*TNFRSF13C*	3
CD38	other	*IFNGR1*	6	*PKHD1*	other	*TNFRSF17*	other
CD3D	1	*IFNGR2*	6	*PLAU*	other	*TNFRSF18*	other
CD3E	1	*IFNLR1*	other	*PLAUR*	other	*TNFRSF1A*	7
CD3G	1	*IGHM*	3	*PLCG1*	other	*TNFRSF4*	1
CD40	1	*IGHMBP2*	other	*PLCG2*	7	*TNFRSF8*	other
CD40LG	1	*IGKC*	3	*PLD1*	other	*TNFSF10*	other
CD46	8	*IGLL1*	3	*PLEKHM1*	6	*TNFSF11*	6
CD5	other	*IKBKB*	1	*PLK1*	other	*TNFSF12*	3
CD55	8	*IKBKG*	2	*PLXNB1*	other	*TNFSF12-TNFSF13*	3
CD59	8	*IKZF1*	3	*PMM2*	other	*TNFSF13B*	other

CD70	4	*IL10*	4	*PMS2*	2	*TNIP1*	other
CD72	other	*IL10RA*	4	*PNP*	2	*TP53*	other
CD74	other	*IL10RB*	4	*PNPLA4*	other	*TP53AIP1*	other
CD79A	3	*IL12B*	6	*PNPT1*	other	*TPP1*	2
CD79B	3	*IL12RB1*	6	*POLA1*	7	*TPP2*	4
CD81	3	*IL17A*	9	*POLE*	2	*TRAC*	1
CD84	other	*IL17F*	6	*POLE2*	2	*TRAF3*	6
CD8A	1	*IL17RA*	6	*PPM1A*	other	*TRAF3IP2*	6
CD93	other	*IL17RC*	6	*PPP2R3B*	other	*TRAF6*	other
CDCA7	2	*IL1B*	other	*PRF1*	4	*TRAFD1*	other
CDKN1B	other	*IL1RN*	7	*PRKCB*	other	*TREX1*	7
CEBPB	other	*IL21*	1	*PRKCD*	4	*TRIM25*	other
CEBPE	5	*IL21R*	1	*PRKCH*	other	*TRIM37*	other
CENPM	other	*IL22*	9	*PRKDC*	1	*TRNT1*	3
CFB	8	*IL2RA*	4	*PRKN*	other	*TRPM2*	other
CFD	8	*IL2RG*	1	*PROC*	other	*TTC37*	3
CFH	8, 9	*IL3*	other	*PRRC2A*	other	*TTC7A*	2
CFHR1	8	*IL36RN*	7	*PSEN1*	6	*TYK2*	6
CFHR2	8	*IL4*	other	*PSENEN*	6	*UBD*	other
CFHR3	8	*IL4R*	other	*PSMA7*	other	*UBE2N*	other
CFHR4	8	*IL6*	9	*PSMB8*	7	*UNC119*	other
CFHR5	8	*IL7*	other	*PSTPIP1*	7	*UNC13D*	4
CFI	8	*IL7R*	1	*PTEN*	3	*UNC93B1*	6
CFP	8	*INO80*	3	*PTPN2*	other	*UNG*	3
CFTR	5	*INPP5D*	other	*PTPRC*	1	*USB1*	5
CHD2	other	*IRAK1*	6	*PTPRN2*	other	*USP15*	other
CHD7	2	*IRAK4*	6	*PURA*	other	*USP18*	7
CHEK1	other	*IRF2BP2*	3	*RAB27A*	4	*USP20*	other
CIITA	1	*IRF3*	6	*RAB37*	other	*USP21*	other
CLCN7	6	*IRF7*	6	*RAB5A*	other	*USP25*	other
CLEC16A	other	*IRF8*	6	*RAB5B*	other	*USP3*	other
CLPB	5	*IRGM*	other	*RAB5C*	other	*VAPA*	other
COG4	other	*ISG15*	6	*RAB7A*	other	*VAV1*	other
COG6	other	*ITCH*	4	*RABGEF1*	other	*VCP*	other
COMMD6	other	*ITGAM*	other	*RAC2*	5	*VDAC1*	other
COPA	7	*ITGB2*	5	*RAD50*	other	*VDR*	other
CORO1A	1	*ITK*	4	*RAD51*	other	*VEGFA*	other
CR2	3	*ITPKB*	other	*RAG1*	1	*VPS13B*	5
CRADD	other	*ITSN1*	other	*RAG2*	1	*VPS45*	5

CRTC3	other	*ITSN2*	other	*RANBP2*	6	*VSTM1*	other
CSF2	9	*JAGN1*	5	*RASGRP1*	4	*VWA2*	other
CSF2RA	5	*JAK1*	4, 6	*RBCK1*	2	*WAS*	2, 5
CSF2RB	5	*JAK3*	1	*RBFOX1*	other	*WASHC5*	other
CSF3R	5	*JMY*	other	*RCC1*	other	*WDR1*	5
CTC1	2	*JUN*	other	*RELA*	other	*WEE1*	other
CTLA4	4	*KANK1*	other	*RELB*	1	*WIPF1*	2
CTPS1	4	*KAT6B*	other	*RFX5*	1	*WRAP53*	2
CTSC	5	*KCTD7*	other	*RFXANK*	1	*XAF1*	other
CX3CR1	other	*KDM6A*	2	*RFXAP*	1	*XIAP*	4
CXCL1	other	*KITLG*	other	*RGCC*	other	*YBX1*	other
CXCL10	other	*KMT2D*	2	*RHOH*	1	*YWHAZ*	other
CXCL12	other	*KRAS*	9	*RHOQ*	other	*ZAP70*	1, 4
CXCL5	other	*LAMTOR2*	5	*RIPK1*	other	*ZBTB24*	2
CXCL8	other						

* International Union of Immunological Societies (IUIS) genes causing inborn errors of immunity were curated from Picard C. et al. J Clin Immunol. 2018 Jan;38(1):96-128 (PMID 29226302), Bousfiha A. et al. J Clin Immunol. 2018 Jan;38(1):129-143 (PMID 29226301). IUIS table numbers reference the Picard et al. 2018 table(s) in which the gene is found and other indicates an immune function gene curated from other sources (see Methods for details).

SUPPLEMENTARY TABLE 2 | Variant burden filtering criteria and results for 669 immune function genes or all genes*

Filter	Selection	669 het EUR		669 het AFR		669 het EUR+AFR	
		Dis variants	Rep variants	Dis variants	Rep variants	Dis variants	Rep variants
none	none	16496	20785	16496	20785	16496	20785
IMPACT	HIGH, MODERATE	2547	3127	2547	3127	2547	3127
gnomAD frequency	<= 0.05	1437	1956	1437	1956	1437	1956
PML_ALT [EUR, AFR, EUR+AFR]	>= 2, >= 1, >= 3	181	298	653	843	99	196
OR	> 1	170	243	642	783	99	161
p-value	<= 0.1	67	73	323	360	53	68
Overlap Dis Rep		8		9		9	
Technical rank = 1		7		9		9	
Biology rank = 1		7		9		9	
>= 1 EUR and AFR and >= 1 Dis and Rep		n/a		n/a		1	

Filter	Selection	All het EUR		All het AFR		All het EUR+AFR	
		Dis variants	Rep variants	Dis variants	Rep variants	Dis variants	Rep variants
none	none	338213	431195	338213	431195	338213	431195
IMPACT	HIGH, MODERATE	65712	84726	65712	84726	65712	84726
gnomAD frequency	<= 0.05	33414	50479	33414	50479	33414	50479
PML_ALT [EUR, AFR, EUR+AFR]	>= 3, >= 1, >= 3	1271	3672	15884	21907	2057	5105
OR	>1	1271	3187	15702	20291	363	484
p-value [EUR, AFR, EUR+AFR]	<= 0.05, <= 0.005, <= 0.01	367	582	1685	1528	363	484
Overlap Dis Rep		22		15		13	
Technical rank = 1		7		10		4	
Biology rank = 1		2		1		0	

* Variant numbers in the Dis and Rep cohorts are identical for EUR, AFR, and EUR+AFR filtered subgroups until an ethnic specific filter (PML_ALT) is applied

SUPPLEMENTARY TABLE 3 | Comparison of statistical analyses for the combined PML cohorts using four control data sets.*

Variant (hg19)	Ethnicity	PML cases	gnomAD GE+GG subjects	p-value	OR	gnomAD GE subjects	p-value	OR	gnomAD GG subjects	p-value	OR	TGP Exome subjects	p-value	OR
22-23915745-G-A	EUR	3/136	0/48/64410	1.77E-04	30.23	0/42/56703	1.77E-04	30.42	0/6/7707	3.97E-04	28.88	0/0/436	1.32E-02	22.89
6-30673359-T-G	EUR	8/136	0/347/60562	1.55E-06	10.85	0/302/54608	1.21E-06	11.24	0/45/5954	1.91E-05	8.20	0/4/426	1.98E-03	6.57
19-7712287-G-C	EUR	4/136	0/311/63507	4.81E-03	6.16	0/267/55798	4.46E-03	6.30	0/44/7709	9.32E-03	5.28	0/1/414	1.46E-02	12.45
9-137779251-G-A	EUR	6/136	2/505/64168	8.21E-04	5.79	1/461/56457	9.85E-04	5.59	1/44/7711	2.30E-04	7.86	0/3/435	7.30E-03	6.62
22-35806756-G-A	EUR	7/136	7/736/64588	1.10E-03	4.66	7/665/56875	1.28E-03	4.54	0/71/7713	3.81E-04	5.84	0/5/436	9.97E-03	4.66
22-23915583-T-C	EUR	5/136	0/541/64515	6.14E-03	4.51	0/446/56811	4.70E-03	4.82	0/95/7704	2.96E-02	3.06	0/4/434	3.93E-02	4.09
2-163136505-C-G	EUR	11/136	6/1367/64143	1.86E-04	4.02	6/1225/56438	2.19E-04	3.95	0/142/7705	6.53E-05	4.69	0/15/436	3.23E-02	2.47
16-81939089-T-C	EUR	5/136	3/610/64302	1.03E-02	3.97	3/533/56593	1.00E-02	3.99	0/77/7709	1.37E-02	3.78	0/4/277	1.63E-01	2.60
16-81942175-A-G	EUR	6/136	2/934/64000	1.56E-02	3.11	2/831/56290	1.64E-02	3.07	0/103/7710	1.15E-02	3.41	0/11/279	7.97E-01	1.12
1-160769595-AG-A	AFR	2/49	0/0/12479	1.50E-05	1313.63	0/0/8123	3.52E-05	855.11	0/0/4356	1.21E-04	458.58	0/0/440	9.86E-03	46.37
19-48643270-C-T	AFR	2/49	0/10/12484	9.64E-04	52.87	0/1/8126	1.05E-04	339.72	0/9/4358	6.25E-03	20.49	0/0/440	9.86E-03	46.37
6-51798908-C-T	AFR	3/49	0/17/12485	6.11E-05	47.68	0/15/8127	1.55E-04	35.15	0/2/4358	1.27E-05	140.77	0/2/440	8.21E-03	14.12
21-45708278-G-A	AFR	2/49	0/23/12433	4.28E-03	22.94	0/17/8079	5.70E-03	20.14	0/6/4354	3.26E-03	30.67	0/3/440	8.09E-02	6.15
1-92946625-G-C	AFR	2/49	0/30/12105	7.31E-03	17.11	0/19/7756	7.51E-03	17.29	0/11/4349	8.77E-03	16.73	0/0/367	1.36E-02	38.68
6-3015818-G-A	AFR	3/49	0/54/12484	1.42E-03	15.00	0/39/8128	1.97E-03	13.51	0/15/4356	9.39E-04	18.81	0/1/440	3.53E-03	28.19
1-57409459-C-A	AFR	2/49	0/38/12483	1.06E-02	13.93	0/28/8128	1.38E-02	12.29	0/10/4355	7.46E-03	18.42	0/0/440	9.86E-03	46.37
1-196918605-A-G	AFR	3/49	0/58/12384	1.76E-03	13.85	0/37/8027	1.77E-03	14.07	0/21/4357	2.22E-03	13.43	0/0/440	9.51E-04	66.31
3-58191230-G-T	AFR	2/49	0/44/12483	1.39E-02	12.02	0/32/8126	1.75E-02	10.75	0/12/4357	1.01E-02	15.37	0/0/440	9.86E-03	46.37
11-67818269-G-A	AFR	7/49	2/490/12463	3.09E-03	4.05	1/318/8113	3.08E-03	4.07	1/172/4350	3.42E-03	4.02	0/20/438	1.25E-02	3.47
16-81942175-A-G	AFR	5/49	0/194/12067	1.21E-03	6.95	0/128/7719	1.42E-03	6.73	0/66/4348	1.05E-03	7.36	0/4/415	9.82E-04	11.55
16-81942175-A-G	EUR+AFR	11/185	2/1128/76067	1.22E-04	4.19	2/959/64009	1.34E-04	4.15	0/169/12058	8.97E-05	4.45	0/15/694	1.25E-02	2.86

* Allele data for gnomAD (GE+GG, GE, GG) and TGP Exome are reported as homozygotes/heterozygotes/total subjects (allele number/2)

SUPPLEMENTARY TABLE 4 | Functional impact of PML-associated variant burden variants and immune dysfunction evidence

Gene symbol	IUIS Gene[a]	Variant (hg19)	gnomAD[b] AF EUR	gnomAD[b] AF AFR	dbSNP ID	Consequence	Impact	PolyPhen	SIFT	MutationTaster	Immune Dysfunction (PMID)[c]
AIRE	yes	21-45708278-G-A	3.89E-05	9.25E-04	rs148012328	p.Gly197Arg	moderate	probably damaging	tolerated	deleterious	27504588, 29483906
C8B	yes	1-57409459-C-A	6.12E-03	1.52E-03	rs139498867	p.Asp382Tyr	moderate	possibly damaging	deleterious	neutral	22773339
CFHR2	yes	1-196918605-A-G	0.00E+00	2.34E-03	rs148175483	p.Lys27Glu	moderate	benign	tolerated	neutral	22773339, 23830046
DNASE1L3	no	3-58191230-G-T	2.48E-03	1.76E-03	rs12491947	p.Asn96Lys	moderate	benign	tolerated	neutral	28533778, 28990587
FCN2	no	9-137779251-G-A	3.97E-03	6.42E-04	rs76267164	p.Arg311Gln	moderate	probably damaging	deleterious	deleterious	25251245, 28894916
GFI1	yes	1-92946625-G-C	3.53E-03	1.24E-03	rs149914857	p.Pro107Ala	moderate	benign	tolerated	deleterious	26447191, 31004086
IFIH1	yes	2-163136505-C-G	1.08E-02	2.81E-03	rs35337543	splice donor	high	n/a	n/a	deleterious	28606988, 28716935*
IGLL1	yes	22-23915583-T-C	4.19E-03	7.23E-03	rs1064421	p.Asn171Ser	moderate	benign	tolerated	deleterious	31291582
IGLL1	yes	22-23915745-G-A	3.73E-04	5.49E-03	rs143780139	p.Thr117Ile	moderate	benign	deleterious	deleterious	31291582
LIG1	yes	19-48643270-C-T	2.39E-03	4.01E-04	rs3730947	p.Val349Met	moderate	possibly damaging	deleterious	deleterious	30395541
LY9	no	1-160769595-AG-A	6.20E-05	0.00E+00	rs763811636	frameshift	high	n/a	n/a	n/a	30791129
MCM5	no	22-35806756-G-A	5.81E-03	8.41E-04	rs2230933	p.Val258Ile	moderate	possibly damaging	tolerated	deleterious	27414250, 29921932
MDC1	no	6-30673359-T-G	2.87E-03	8.02E-03	rs143258964	p.Thr1201Pro	moderate	benign	tolerated	neutral	29921932, 30453211
NQO2	no	6-3015818-G-A	1.55E-05	2.16E-03	rs148024596	p.Val120Met	moderate	probably damaging	deleterious	deleterious	29934320
PKHD1	no	6-51798908-C-T	2.33E-05	6.81E-04	rs199589074	p.Gly2041Ser	moderate	probably damaging	deleterious	deleterious	27225849*, 29140564
PLCG2	yes	16-81939089-T-C	4.79E-03	7.45E-04	rs187956469	p.Tyr482His	moderate	possibly damaging	tolerated	deleterious	25349203*, 29538758
PLCG2	yes	16-81942175-A-G	7.33E-03	8.04E-03	rs75472618	p.Asn571Ser	moderate	benign	deleterious	deleterious	25349203*, 26206677
STXBP2	yes	19-7712287-G-C	2.45E-03	3.75E-04	rs35490401	p.Arg529Pro	moderate	probably damaging	deleterious	deleterious	24916509, 29599780
TCIRG1	yes	11-67818269-G-A	1.61E-03	1.98E-02	rs75596506	p.Ala826Thr	moderate	benign	tolerated	neutral	30837952

[a] International Union of Immunological Societies (IUIS) genes causing inborn errors of immunity were curated from Picard C. et al. J Clin Immunol. 2018 Jan;38(1):96-128 (PMID 29226302) and Bousfiha A. et al. J Clin Immunol. 2018 Jan;38(1):129-143 (PMID 29226301).

[b] Allele Frequency (AF) in gnomAD subjects (genomes + exomes) for EUR (non-Finnish Europeans) and AFR ethnicities.

[c] PubMed ID (PMID) numbers for example studies or reviews that provide additional supporting evidence of immune dysfunction for the PML-associated genes; PMID marked with an asterisk (*) report on the specific variant.
